# Predicting the Evolution of Lung Squamous Cell Carcinoma In Situ Using Computational Pathology

**DOI:** 10.3390/bioengineering12040377

**Published:** 2025-04-02

**Authors:** Alon Vigdorovits, Gheorghe-Emilian Olteanu, Ovidiu Tica, Andrei Pascalau, Monica Boros, Ovidiu Pop

**Affiliations:** 1 Department of Pathology, Bihor County Clinical Emergency Hospital, 410169 Oradea, Romania; alonvigdorovits@gmail.com (A.V.); ovidiu.tica@gmail.com (O.T.); pascalau.andrei@gmail.com (A.P.); monicadohan76@yahoo.com (M.B.); drovipop@gmail.com (O.P.); 2 Department of Morphological Sciences, Faculty of Medicine and Pharmacy, University of Oradea, 410073 Oradea, Romania; 3 Department of Pathology, British Columbia Cancer Agency, Vancouver, BC V5Z 4E6, Canada

**Keywords:** computational pathology, deep learning, squamous cell carcinoma in situ

## Abstract

Lung squamous cell carcinoma in situ (SCIS) is the preinvasive precursor lesion of lung squamous cell carcinoma (SCC). Only around two-thirds of these lesions progress to invasive cancer, while one-third undergo spontaneous regression, which presents a significant clinical challenge due to the risk of overtreatment. The ability to predict the evolution of SCIS lesions can significantly impact patient management. Our study explores the use of computational pathology in predicting the evolution of SCIS. We used a dataset consisting of 112 H&E-stained whole slide images (WSIs) that were obtained from the Image Data Resource public repository. The dataset corresponded to tumors of patients who underwent biopsies of SCIS lesions and were subsequently followed up by bronchoscopy and CT scans to monitor for progression to SCC. We used this dataset to train two models: a pathomics-based ridge classifier trained on 80 principal components derived from almost 2000 extracted features and a deep convolutional neural network with a modified ResNet18 architecture. The performance of both approaches in predicting progression was assessed. The pathomics-based ridge classifier model obtained an F1-score of 0.77, precision of 0.80, and recall of 0.77. The deep learning model performance was similar, with a WSI-level F1-score of 0.80, precision of 0.71, and recall of 0.90. These findings highlight the potential of computational pathology approaches in providing insights into the evolution of SCIS. Larger datasets will be required in order to train highly accurate models. In the future, computational pathology could be used in predicting outcomes in other preinvasive lesions.

## 1. Introduction

Lung cancer (LC) is the leading cause of cancer mortality, with an estimated 1.8 million deaths per year [1]. Squamous cell carcinoma of the lungs (SCC) accounts for about 20% of all lung cancers and is more heavily associated with smoking than lung adenocarcinoma [2]. It usually occurs in the proximal part of the airway and originates typically from the basal cells of the bronchial mucosa [3]. SCC shows numerous genetic alterations but only a few clinically actionable driver mutations in contrast to lung adenocarcinoma, which has several targetable driver mutations, such as *EGFR*, *ALK*, and *ROS1* [4]. Squamous cell carcinoma in situ (SCIS) is the preinvasive precursor lesion of SCC. Given the fact that 30% of SCIS undergo spontaneous regression, the clinical management of patients that present with SCIS is challenging and often results in overtreatment [5]. Usually, patients have multiple comorbidities, further complicating clinical decisions [6]. Patients with SCIS have significantly worse survival than those with lung adenocarcinoma in situ and a similar prognosis to those with stage IA SCC [7].

In recent years, low-dose helical CT screening seems to offer a promising way to improve survival in SCC [7]. Unfortunately, a CT scan might not always detect preinvasive lesions [5]. Endobronchial photodynamic therapy is the most well-studied treatment modality, with complete response rates varying between 32 and 100% [8]. One epidemiologic study found that lobectomy is the procedure associated with the highest overall survival in these patients [7]. Another group found that lobectomy and wedge resection are equivalent for patients with stage IA non-small cell lung cancer (NSCLC) < 20 mm [9]. The ability to predict which SCIS will progress to SCC would be invaluable to help guide further monitoring and treatment. Previous studies have characterized the molecular profiles of SCIS in order to predict if the lesions will progress to SCC or spontaneously regress [8]. Nonetheless, given the fact that progressive and regressive lesions are indistinguishable from each other, morphology was not directly used to predict progression.

The totality of the features extracted from histopathology images, generally termed pathomics, allows for the analysis of image characteristics associated with certain outcomes in a quantitative manner [10]. Recently, several researchers explored the use of pathomics as a predictive tool [11,12,13]. Extracting all the information contained in these images leads to datasets with hundreds or thousands of variables, which can lead to overfitting when used to train predictive models [14]. Using dimensionality reduction techniques aids in creating datasets that are less prone to overfitting that can still be utilized in training machine learning models [11].

Deep learning (DL) is a subfield of machine learning that uses artificial neural networks (ANNs) in order to learn patterns from highly complex data. ANN are non-linear statistical models that are loosely based on biological neural networks and have achieved tremendous success in various pattern recognition tasks [9]. Medical imaging and especially histopathology are ideal for analysis via DL techniques due to their high information density [15]. Researchers have used DL to classify various tumors, predict molecular alterations directly from H&E-stained images, and estimate survival from histomorphology [16,17,18]. Histopathology images can thus be datamined for a wealth of clinically actionable data, some of which could hold information regarding the natural history of certain lesions. DL approaches allow an end-to-end analysis of images, without the need for feature selection, which can be time-consuming and subject to biases.

In this study, we explore the use of pathomics, as well as end-to-end DL, to predict the evolution of SCIS lesions. Our approach involves extracting approximately 2000 features from WSI, followed by feature normalization and the use of principal component analysis (PCA) as a dimensionality reduction technique. We then perform ridge classification on the derived principal components. We also trained a deep convolutional neural network (DCNN) to perform end-to-end prediction from the same images without the need to extract features and compared the results to those obtained from the pathomics pipeline. To our knowledge, this is the first study that uses a computational pathology approach in an attempt to predict the course of SCIS. This study demonstrates the potential of diverse computational pathology methods in predicting the evolution of preinvasive precursor lesions and may inform future research involving applying these techniques to other similar lesions.

## 2. Materials and Methods

### 2.1. Dataset

**Figure 1:**
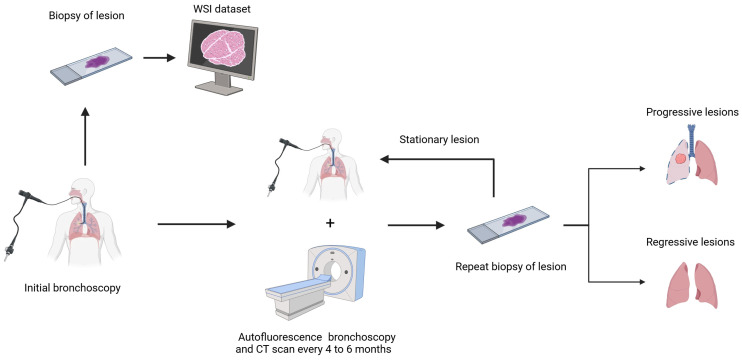
Study protocol.

We used a publicly available WSI dataset from patients with SCIS [19], obtained from the Image Data Resource public repository [20]. This represents the largest cohort of patients with these types of lesions. The cohort consisted of patients diagnosed with SCIS by histopathology before enrolling in the study. Subsequently, patients underwent autofluorescence bronchoscopy and CT scans every 4 to 6 months, with biopsies from the same lesion taken during each bronchoscopy [21]. The dataset consisted of 112 H&E-stained WSI of the initial SCIS biopsy in SVS format. The initial lesions were defined as progressive if subsequent biopsies revealed invasive cancer and regressive if they showed normal respiratory epithelium, metaplasia, or mild/moderate dysplasia (Figure 1). Overall, 68 lesions progressed to invasive cancer, and 44 underwent regression.

### 2.2. Feature Extraction and Dimensionality Reduction

**Figure 2:**
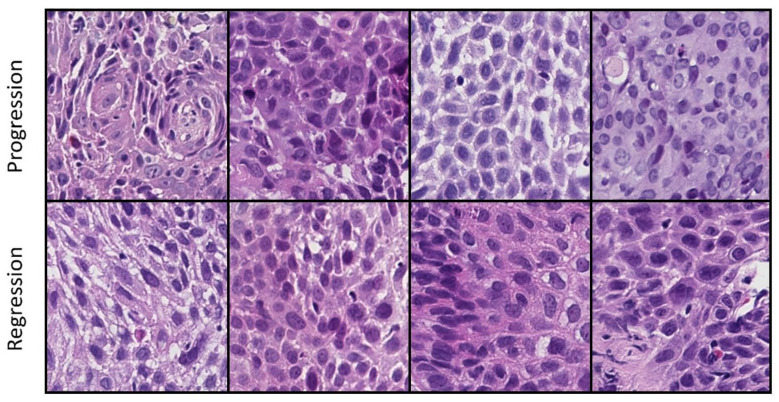
Example images used in the computational analysis.

Initially, in order to prevent data leakage generated by having images from the same patient used for both training and testing, a test dataset consisting of 20% of the WSI was generated by random sampling. Then, regions of interest from each WSI were selected by a thoracic pathologist. Tiling of the regions of interest generated a variable number of 256 × 256 pixel images at 20× magnification from each WSI. Examples of tiles used for the downstream analysis can be seen in Figure 2. The number of tiles per WSI varied depending on the size of the biopsy and the size of the lesion. The final dataset contained 11,130 images. Of these, 7907 images were from lesions that progressed to invasive cancer and 3223 from lesions that regressed. The training dataset had 8800 images, while the test dataset contained 2330 images. A custom automated feature extraction pipeline was built using CellProfiler, version 4.2.6 [22]. The pipeline consisted of multiple steps, including the identification of nuclei and cells, measurement of the color intensity, intensity distribution, texture, granularity, and various other size and area variables. The result of this pipeline consisted of around 2000 features for each image. These measurements were averaged across all tiles extracted from a WSI to obtain WSI-level data. The main categories of measurements were area and shape, perimeter and boundary, texture, granularity, intensity, location, and object count. Examples from each category are highlighted in Table 1. Due to the large number of features and comparatively low number of cases, dimensionality reduction using PCA was performed using the Python module Scikit-learn version 1.5.2 [23]. Before the PCA, each feature was normalized by subtracting the image level mean and dividing by the standard deviation (Z-score normalization). We selected the minimum number of principal components required to explain 95% of the total variance in the data.

The principal components were used as variables for a ridge classifier model that predicted the progression of the lesions. The RidgeClassifier function from Scikit-learn was used, with a maximum of 10,000 iterations to obtain algorithm convergence. Hyperparameter optimization was performed via a grid search strategy using 10-fold cross-validation. The F1-score, precision, recall, and accuracy were the calculated metrics. The F1-score was included in order to deal with the imbalanced classification problem.

### 2.3. Deep Learning Method

The same tiles extracted from WSIs used in the pathomics pipeline were used to train and test the DL model. In order to improve the generalizability of the model and prevent overfitting, various data augmentation techniques such as random vertical flipping, random horizontal flipping, random rotations, and color jitter were applied to the images prior to training. The training dataset was used to train the ResNet18 network architecture [24]. Our DL library of choice was PyTorch version 2.4.0 [25]. A training/validation split of 80/20 was used, with the model hyperparameters being tuned according to the results obtained on the validation set. The fully connected portion of the ResNet18 model contained a dropout layer with a dropout frequency rate of 0.7 in order to prevent overfitting. The model was initially pretrained on ImageNet and then fine-tuned on the training data using a stochastic gradient descent optimization algorithm with a learning rate of 10^−3^ for 5 epochs; after which, the learning rate was decreased to 10^−4^ for another 5 epochs [26]. At this point, the model had already reached convergence. The per-tile performance metrics used were F1-score, AUC (area under the receiver operating characteristic curve), recall (sensitivity), and precision. After hyperparameter optimization, the best candidate model was selected, and the performance was evaluated using the held-out test dataset. The mean and standard deviation obtained across 5 random seeds were reported. In order to obtain WSI-level predictions, we added a module that performed a probability weighted average of the tiles in each WSI. The performance metrics were then calculated at the WSI level. Gradient-weighted Class Activation Maps (Grad-CAMs) were generated from the final convolutional layers of the trained neural network. For each analyzed patch, gradients were computed via backpropagation to identify regions that are associated with model classification. All of the code used for image preparation, as well as model training and testing, is available at https://github.com/ohalon/SCISEvo (accessed on 13 March 2025).

## 3. Results

### 3.1. Pathomics Pipeline

**Figure 3:**
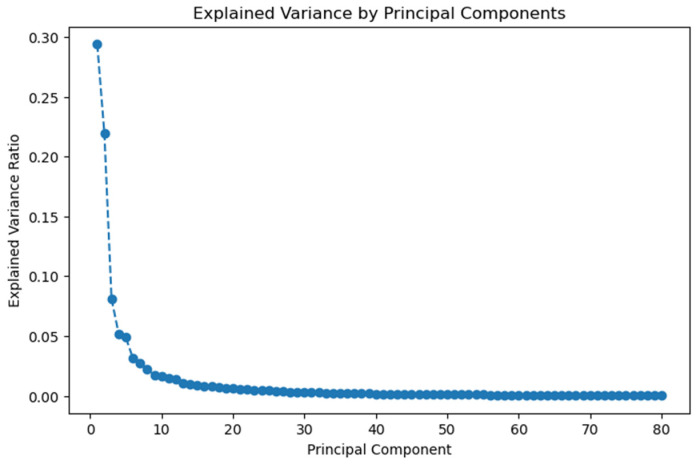
Explained variance of the principal components.

After feature extraction and manual curation, the dataset consisted of 1965 variables. The PCA performed on this dataset resulted in the extraction of 80 principal components, which were used for the downstream analysis. These components cumulatively explain 95% of the total variance in the data. The relationship between the main principal components and the explained variance can be seen using the scree plot in Figure 3.

**Figure 4:**
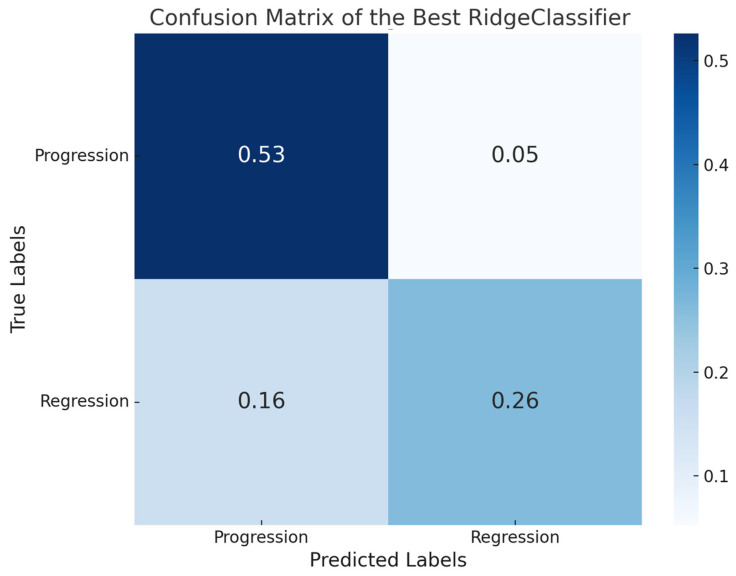
Confusion matrix for the ridge classifier.

Following 10-fold cross-validation for eight candidate models identified through a grid search, a total of 80 model fits were performed. The most accurate model reached an F1-score of 0.77, a precision of 0.80, a recall of 0.77, and an accuracy of 0.79 on the held-out test dataset. The confusion matrix of the best performing ridge classifier is shown in Figure 4.

### 3.2. Deep Learning Pipeline

**Figure 5:**
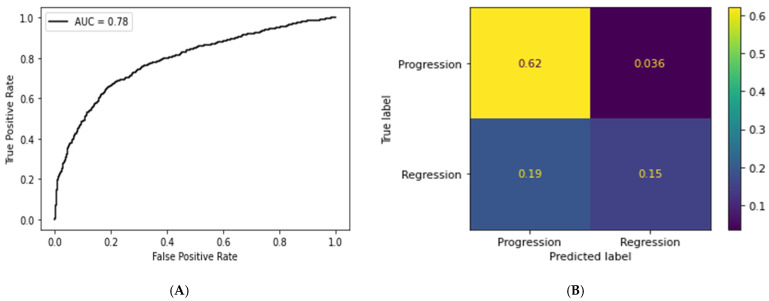
(**A**) Tile-level ROC curve. (**B**) Tile-level confusion matrix.

The deep learning model achieved a mean per-tile AUC of 0.78 with a standard deviation (SD) of 0.01 (Figure 5A). The selected threshold probability for progression was 0.5. The DCNN model also yielded the following tile-level metrics: F1-score of 0.84 (SD = 0.05), recall (sensitivity) of 0.94 (SD = 0.01), and precision of 0.76 (SD = 0.007). After probability-weighted averaging, we obtained a WSI-level F1-score of 0.80 (SD = 0.03), sensitivity of 0.90 (SD = 0.008), and precision of 0.71 (SD = 0.005) (Table 2). Figure 5B represents the tile-level confusion matrix obtained from the classification results.

**Figure 6:**
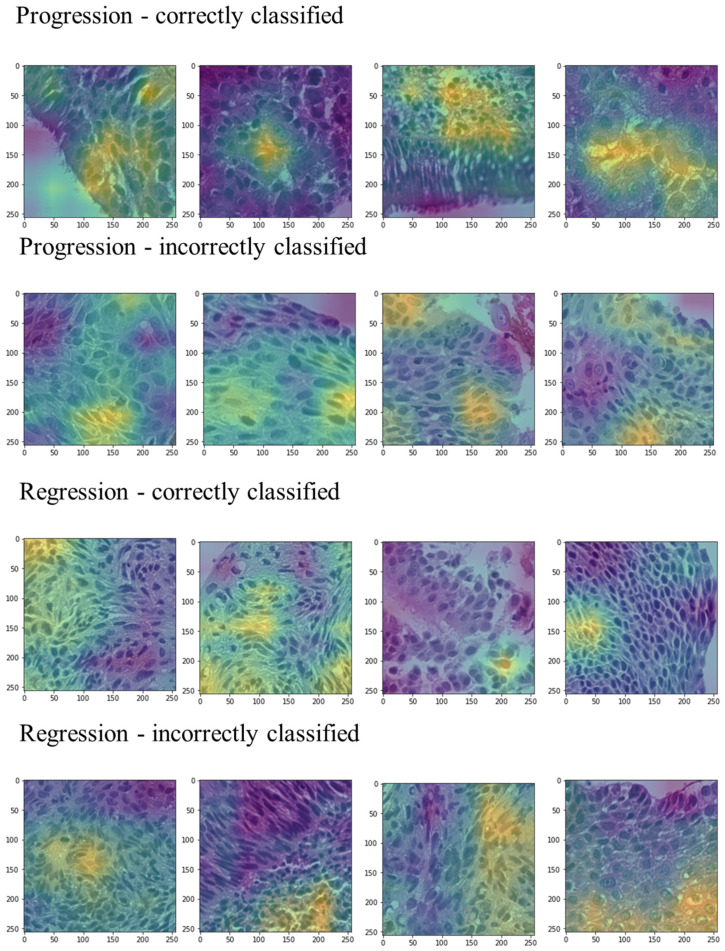
Example Grad-CAM heatmaps of correctly and incorrectly classified cases from both classes. Bright yellow indicates areas that have a significant contribution to model prediction.

The Grad-CAM heatmaps generated from image patches (Figure 6) highlight areas of increased activation contributing significantly to the classification decisions of the trained deep learning model. Bright yellow indicates regions that strongly influenced the predictions.

## 4. Discussion

Our study highlights the potential of multiple computational pathology approaches to tackle a seldom approached predictive challenge. Both pathomics and feature extraction-based pipelines, as well as end-to-end “black box” DL model architectures, were used to predict the evolution of SCIS lesions. Our feature extraction pipeline captured almost 2000 sub-visual but interpretable features with sub-cellular resolution from WSI regions of interest that were selected by a thoracic pathologist. The creation of the pipeline and curating features requires more time and expertise but provides enhanced explainability and insight, while the DL approach only requires region of interest annotation and tiling at the cost of reduced explainability.

With improved access to care, as well as technological advancements, bronchoscopy has increased our capacity to detect SCIS. Up to 40% of these lesions can be detected using white light reflectance bronchoscopy (WLB). Autofluorescence bronchoscopy offers increased sensitivity of up to 85% when associated with WLB [27,28]. Optical coherence tomography with infrared illumination could offer a spatial resolution down to 3 µm and a penetration depth of 2 mm [29]. These advances have the potential to greatly increase the number of SCIS diagnoses made in the future. The inability to predict the clinical evolution of SCIS generates issues in the management of these patients. Considering the fact that 30% of SCIS regress and never become invasive carcinomas, overtreating these lesions is a persistent problem that leads to increased morbidity and mortality. Another issue is represented by the slow course of progression, with studies showing that patients with SCIS have a 5-year survival of over 90% [8]. Currently, there are no histopathological criteria to assess the progressive potential of SCIS. Even though previous studies have managed to find molecular markers that are highly predictive for progression, complex molecular assays are difficult to implement to scale, especially in the developing world.

Both computational approaches in our study provided similar results. The ridge classification model, trained using 80 principal components generated from the extracted features, achieved an F1-score of 0.77, an accuracy of 0.79, a precision of 0.80, and a recall of 0.77 on the test dataset, which is comparable to the WSI-level results of the DL model, which reached an F1-score of 0.80, an accuracy of 0.75, a precision of 0.71, and a recall of 0.90.

Pathomics-based computational pathology with feature extraction from WSIs provides a plethora of image features, such as various measures of area and shape, boundaries, granularity, centrality, texture, and object counts. The large number of variables extracted predisposes models to overfitting issues. Thus, expert knowledge is required to select and cluster features, which can be done in conjunction with dimensionality reduction approaches such as PCA. The main disadvantage of DL models is reduced explainability, which is a key aspect to take into account when considering the adoption of artificial intelligence (AI) in pathology. This can be mitigated by using certain methods such as Grad-CAM heatmaps or attention mechanisms. In the future, the creation of feedback loops between the pathologist and the models used will be vital for increasing the accuracy and decision-making transparency and providing pathologist-centered AI models. Various methods of visualizing the way DL models generate their predictions could also aid pathologists in discovering new morphologic clues.

The study has certain limitations. Our dataset only consisted of 112 WSIs, out of which we managed to extract 11,130 images. Extracting thousands of features from images creates datasets with very high dimensionality, which results in predictive models that frequently face issues with overfitting unless provided with more data. DL models also require large datasets, and their performance increases with the amount of training data [30]. However, sizable cohorts of patients with SCIS are very rare, as they require performing invasive procedures, as well as a long follow-up period. Collaboration and pooling of smaller in-house cohorts would be needed to generate larger datasets on which even more accurate models could be trained.

## 5. Conclusions

To our knowledge, this study is the first to explore the use of computational pathology in predicting the evolution of SCIS. We investigated a pathomics-based approach that utilizes a large number of explainable features extracted from WSIs, as well as a DP-based end-to-end methodology; both approaches yielded similar results. These findings suggest that computational pathology could be applied to predict other preinvasive precursor lesions in various organs using only H&E-stained WSIs. As more and more countries adopt screening programs and larger training databases of these lesions become available, computational pathology has the potential to become part of the screening toolkit, offering a high-throughput and low-cost method of providing patients and clinicians with enhanced prognostic information.

## Figures and Tables

**Table 1:** Feature categories and examples.

Feature Category	Feature Examples
Area and Shape	Mean cytoplasmic area Median nuclear compactness Mean cytoplasmic form factor
Perimeter and Boundary	Mean cytoplasmic perimeter area Mean nuclear bounding box area Mean nuclear ferret diameter
Texture	Mean cytoplasm eosin contrast Mean nuclear angular second moment Median cytoplasm hematoxylin texture correlation
Granularity	Eosin granularity Mean nuclear hematoxylin granularity Median eosin cytoplasm granularity
Intensity	Eosin intensity Mean nuclear hematoxylin intensity Median cytoplasm hematoxylin intensity
Location	Mean cytoplasm center location Mean hematoxylin center location Median cytoplasm mass center
Object Counts	Cytoplasmic object count Nuclear count

**Table 2:** Tile- and WSI-level results.

	Accuracy	F1-Score	Sensitivity	Precision
Tile	0.77	0.84	0.94	0.76
WSI	0.75	0.80	0.90	0.71

## Data Availability

The data used in the study are available in the Image Data Resource public repository.
